# Bioactive Plant Compounds as Alternatives Against Antifungal Resistance in the *Candida* Strains

**DOI:** 10.3390/pharmaceutics17060687

**Published:** 2025-05-23

**Authors:** Thainá dos Santos Dantas, Janaina Carla Barbosa Machado, Magda Rhayanny Assunção Ferreira, Luiz Alberto Lira Soares

**Affiliations:** 1 Postgraduate Program in Therapeutic Innovation, Federal University of Pernambuco, Recife 50670-901, Brazil; thaina.dantas@ufpe.br; 2 Postgraduate Program in Pharmaceutical Sciences, Federal University of Pernambuco, Recife 50670-901, Brazil; janaina.machado@ufpe.br

**Keywords:** fungal infections, antifungal resistance, bioactive compound, phytochemical, medicinal plants

## Abstract

The pathogenicity of *Candida* spp. poses a persistent challenge, particularly in hospital environments where these species proliferate and cause opportunistic infections. Many strains have developed resistance to commonly used antifungal agents, including azoles, polyenes, and echinocandins, complicating treatment, especially in immuno-compromised patients. Understanding the mechanisms underlying antifungal resistance, such as mutations in genes involved in ergosterol biosynthesis, efflux pump activity, and enzymatic pathways, is crucial for developing targeted interventions. Given the challenges associated with discovering new antifungal agents, medicinal plants have emerged as a promising source of bioactive compounds with anti-*Candida* activity. Secondary metabolites, including terpenoids, alkaloids, flavonoids, and tannins, exhibit various mechanisms of action, such as biofilm inhibition, membrane disruption, and oxidative stress induction. However, challenges such as extract standardization, and the lack of clinical studies continue to limit their therapeutic application. This review underscores the potential of medicinal plants as complementary or alternative strategies to conventional antifungal therapies, emphasizing the need for multidisciplinary research to overcome these hurdles and harness the therapeutic potential of natural products.

## 1. Introduction

Fungal infections have become a serious public health concern, affecting approximately 6 to 55 million people worldwide with life-threatening diseases, particularly those caused by opportunistic fungi, which complicate treatment [1]. Moreover, several studies have highlighted the impact of these infections, which exacerbate the conditions of patients with pre-existing illnesses or those hospitalized, making them one of the main challenges in clinical practice. Key opportunistic fungal species include *Candida* spp., *Sporothrix* spp., *Aspergillus* spp., and *Cryptococcus* spp., which account have mortality rates of approximately 90–100% if left untreated, causing diseases of the skin, lungs, vagina, bloodstream, and meninges [2,3]. These infections can be classified as superficial, subcutaneous, or systemic, involving a variety of species that exhibit both yeast-like and filamentous forms, each with varying degrees of virulence [4,5].

The growing resistance of fungi to conventional antifungal drugs underscores the urgent need for new therapeutic alternatives. Among these, natural products, particularly plant-derived compounds, have gained significant attention due to their unique mechanisms of action and structural diversity [6,7]. Secondary metabolites produced by plants, such as terpenoids, flavonoids, and alkaloids, serve as natural defenses against external threats and exhibit various pharmacological activities, including potent antifungal effects. Their complex structures enable them to interact with fungal cells in novel ways, offering potential solutions to overcome resistance. Several studies have highlighted the antifungal properties of various plant species, particularly their activity against *Candida* spp., demonstrating promising results even against resistant strains [8,9]. This positions plant-based natural products as a viable alternative to conventional antifungal resistance.

Therefore, there is an increasing scientific interest in medicinal plants with antifungal activity, particularly against *Candida* species. In this context, the article search was conducted between November and December 2024, using ScienceDirect and PubMed databases. The following descriptors were applied: “natural products AND *Candida*”, “*Candida* spp.”, “resistance mechanisms”, and “antifungals AND *Candida*”, with most studies selected from the past five years. This review examines the resistance mechanisms associated with current antifungal agents and explores the potential of phytochemicals as alternative candidates for developing new drugs targeting fungal cells.

## 2. *Candida* spp.

**Figure 1:**
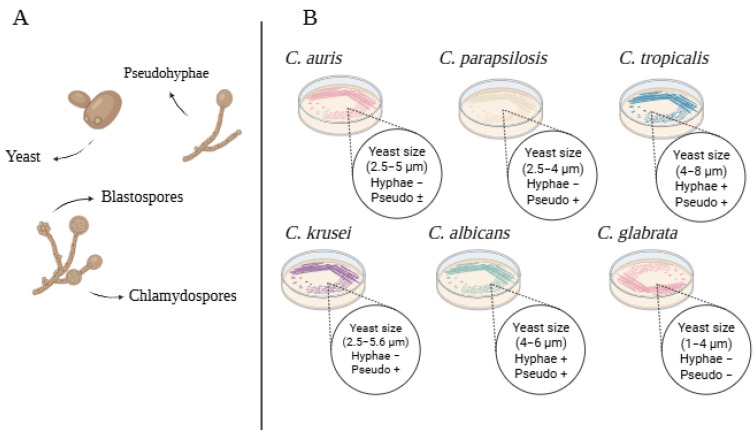
Schematic representation of different *Candida* species (created in BioRender. Thainá dos Santos Dantas. (2025) https://app.biorender.com/illustrations/6818cb4005105a81ae8b5da0). (**A**) Morphological forms of *Candida*; (**B**) growth characteristics on Chromagar, of *Candida* (yeast size, hyphae, and pseudohyphae formation).

The genus *Candida* includes a diverse range of species that exhibit various morphological forms, such as unicellular yeast cells that reproduce through budding, and multicellular forms like hyphae and pseudohyphae (Figure 1). These structural adaptations give *Candida* species resistance mechanisms to survive in diverse environments. Among the species of greatest clinical relevance are *Candida albicans*, the primary cause of systemic infections in immunocompromised patients, as well as *C. tropicalis*, *C. glabrata*, *C. parapsilosis*, *C. krusei*, and, more recently *C. auris* [6].

Candidiasis can manifest as superficial lesions on mucosal surfaces, such as the mouth and vulvovaginal areas, or on the skin, causing symptoms like pruritus, pain, and discharge. In more severe cases, candidiasis may progress to systemic infections, with the fungus invading tissues and entering the bloodstream, thereby impairing organ function. The severity of these infections depends on the species involved and their virulence factors, which facilitate fungal proliferation and worsen the disease course. Virulence factors, such as biofilm formation and enzyme production, often contribute to the development of resistance mechanisms, exacerbating the scarcity of effective therapeutic options [8,10,11].

*Candida* species are opportunistic pathogens that exhibit several virulence traits, including adherence capabilities, morphological polymorphism, phenotypic variability, toxin production, enzymatic activity, and biofilm formation. These traits play a crucial role in the pathogenicity and differentiation of *Candida* species. Identification is commonly performed using clinical sample cultures, microscopy, fermentation and biochemical tests, and molecular techniques such as PCR (polymerase chain reaction) [12]. For example, adhesins responsible for cellular adhesion are encoded by different genes in *Candida* species. In *C. albicans*, cellular adhesion is mediated by the ALS (agglutinin-like sequence) family, which enhances its adhesion potential compared to other species, such as *C. tropicalis* and *C. glabrata*, where adhesion is encoded by EPA (epithelial adhesin) [13]. Furthermore, Ramos et al. [14] observed that the isolates with the highest biofilm formation and metabolic activity were from *C. tropicalis*, followed by isolates of *C. krusei*.

## 3. Antifungal Agents and Resistance Mechanisms

In recent years, the increasing prevalence of diseases exacerbated by candidiasis has motivated extensive research into therapies targeting *Candida* spp. Various classes of antifungal drugs with distinct mechanisms of action have been developed and incorporated into clinical practice. The main classes of antifungal agents include polyenes, azoles, allylamines, echinocandins, and pyrimidines, with four of these azoles, polyenes, echinocandins, and pyrimidines standing out due to their proven efficacy and widespread clinical use in the treatment of candidiasis [15]. These classes were selected for detailed analysis in this review based on their distinct pharmacological profiles, broad spectrum of activity, and clinical relevance in the management of *Candida* infections. Although allylamines also act by inhibiting ergosterol biosynthesis, their antifungal spectrum is more effective against dermatophytes, and they are primarily indicated for cutaneous and superficial fungal infections [4,16].

While these drugs are effective, their use requires caution, as some compounds also bind to human steroids, leading to adverse effects such as cellular toxicity. Additionally, the indiscriminate and continuous use of antifungal treatments has driven the evolution of resistance mechanisms, allowing fungi to evade the effects of these drugs [17,18].

The growing prevalence of antifungal resistance has prompted significant research into alternative therapies, including comparative studies and drug combinations. *Candida* resistance can occur through various strategies, such as drug target modification, overexpression of efflux pumps that expel the drug from the cell, or changes in cell membrane permeability that inhibit drug entry. Studies have also identified fungi lacking ergosterol or carrying mutations in specific genes as key resistance mechanisms [19,20]. In Table 1, the various mechanisms of resistance of antifungal agents used against *Candida* spp. are shown.

### 3.1. Azoles

**Figure 2:**
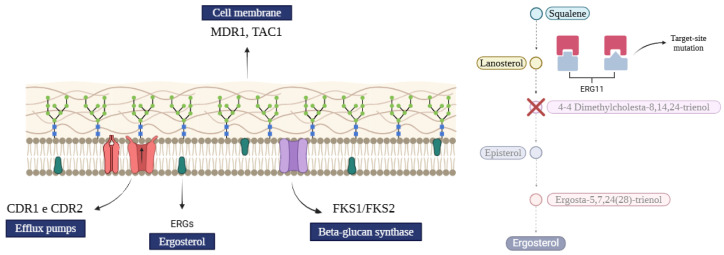
Schematic representation of the primary pharmacological targets of antifungal drug classes (Created in BioRender. Thainá dos Santos Dantas. (2025) https://app.biorender.com/illustrations/61b20227de229900a4dcec99 and https://app.biorender.com/illustrations/682e3b99a2ab9dda551d0c4a).

Azoles are a group of antifungal drugs characterized by the presence of nitrogen atoms in their cyclic carbon structure, with a primarily fungistatic action. Key representatives of this class include fluconazole, miconazole, clotrimazole, and voriconazole. These drugs target the fungal cell membrane by inhibiting ergosterol synthesis, a crucial component for maintaining membrane integrity and stability (Figure 2). The disruption of ergosterol synthesis results in membrane destabilization and ultimately inhibits fungal growth [11,29].

The mechanism of action involves the inhibition of 14α-demethylase, an enzyme essential for converting lanosterol into ergosterol. By blocking this enzyme, azoles cause an accumulation of toxic sterol intermediates, leading to membrane dysfunction and altered permeability. Studies suggest that azoles target the heme protein by binding to the iron atom in its active site through the nitrogen atom in the azole ring [29].

However, resistance to azoles, particularly fluconazole, is widespread among *Candida* spp. Resistance mechanisms include genetic alterations that reduce the drugs’ efficacy. Efflux pumps encoded by the *CDR1* and *CDR2* genes, regulated by transcription factors such as *TAC1* and *MDR1*, actively expel azoles from the fungal cell, diminishing their effectiveness [21,22,30]. Another resistance mechanism involves mutations in the lanosterol 14α-demethylase enzyme, encoded by the *ERG11* gene, which decreases azole binding affinity. Additionally, mutations in the ERG3 gene, involved in ergosterol biosynthesis, have been shown to redirect the metabolic pathway, preventing the accumulation of toxic intermediates. This alternative pathway allows the fungal cell to maintain functionality and avoid cellular damage, rendering azoles ineffective [23,26].

Most *Candida* species share common resistance mechanisms; however, certain genes are more prominently expressed in some species compared to others. For instance, *C. albicans* exhibits a higher expression of *CDR1*, triggered by the transcription factor *TAC1*, which constitutes a primary pathway for azole resistance in this species. This contrasts with *C. auris*, where *CDR1* expression is not similarly affected [31]. As reported by [22], blood isolates of *C. albicans* not only exhibited *CDR1* expression but also showed increased expression of *CDR2*. Similarly, *C. glabrata* isolates demonstrated higher expression of the *CDR1* gene. In *C. tropicalis* isolates, 6 out of 17 samples expressed *MDR1* genes, while no expression of *CDR2* was detected. Supporting these findings, the studies by [23], on fluconazole-resistant *C. glabrata* isolates from different continents revealed an upregulation of efflux pump genes (*CDR1* and *CDR2*). It is important to note that these mechanisms may vary depending on the region from which the *Candida* isolates are obtained, potentially leading to mutations with varying intensities and specificities.

### 3.2. Polyenes

Polyenes are a class of antifungal agents characterized by their specific interaction with ergosterol (Figure 2), a vital component of the fungal cell membrane. This interaction disrupts the membrane’s structural integrity, rendering it susceptible to damage and ultimately leading to cell lysis [18]. The primary representatives of this class are amphotericin B and nystatin. While both exhibit fungicidal activity, amphotericin B is more effective against a wide range of fungi and is commonly used to treat systemic infections, whereas nystatin is primarily employed for topical treatment of cutaneous infections [19,32].

The mechanism of action of polyenes involves their affinity for sterols in fungal cells, specifically ergosterol. This binding triggers the release of hydroxyl radicals into the cell, disrupting membrane functionality and causing its rupture. Additionally, polyenes form pores in the fungal cell membrane, leading to an efflux of ions and essential intracellular components, which further compromises fungal survival [4,5].

Resistance to this class of antifungal agents is rare; however, some *Candida* species have been reported to exhibit mutations that reduce the antifungal efficacy of amphotericin B. Genes involved in ergosterol biosynthesis indicate that *Candida* strains, particularly *C. albicans* and *C. glabrata*, and show resistance to amphotericin B due to mutations in *ERG3*, *ERG5*, *ERG6*, and *ERG11*. Additionally, biofilm formation represents another resistance mechanism, potentially correlated with the overexpression of the *ALS3* and *FKS1* genes in *C. albicans*. These genes encode glycoproteins located on fungal cells, enhancing biofilm formation and contributing to resistance [12,15,25].

### 3.3. Echinocandins

Echinocandins are a class of antifungal agents that target the fungal cell wall by inhibiting the enzyme β-1,3-D-glucan synthase (Figure 2). This inhibition compromises cell wall integrity, leading to cellular damage and ultimately fungal cell death. Key drugs in this class include caspofungin, micafungin, and anidulafungin, which are commonly used to treat invasive fungal infections, particularly in immunocompromised patients [18,33]. The mechanism of action of echinocandins involves disrupting the protein complex responsible for producing β-1,3-glucans. This process is regulated by GTP-binding peptides, *Fks1p* and *Fks2p*. However, the precise binding site of echinocandins on these proteins remains unclear, suggesting that additional mechanisms or alternative targets within the β-1,3-glucan synthesis pathway might be involved [4,29].

Although echinocandins were developed to address resistant fungal strains, cases of resistance have been reported. Studies indicate that mutations in the FKS1 and FKS2 genes can alter regions critical to the enzyme β-1,3-D-glucan synthase, reducing drug efficacy [24,26]. These mutations vary among different *Candida* species, resulting in varying levels of resistance [17,29]. Mutations in FKS1 can be found in caspofungin-resistant *C. auris* isolates, as well as in *C. glabrata* strains, which may also include FKS2 mutations. These mutations result in an increased minimum inhibitory concentration (MIC) of the drug. [27,28]. Furthermore, research shows that infections caused by *Candida* isolates carrying *MDR* (multidrug resistance) genes are associated with decreased effectiveness of this drug class [34,35].

### 3.4. Pyrimidines

5-Fluorocytosine (5-FC) is a fluorinated pyrimidine analog with antifungal activity that acts as an antimetabolite, inhibiting nucleic acid synthesis in pathogenic yeasts such as *Candida* spp. and *Cryptococcus neoformans* [11]. Its mechanism of action involves a cascade of enzymatic conversions that ultimately lead to the inhibition of both RNA and DNA synthesis, resulting in fungal cell death. Initially, 5-FC is converted into 5-fluorouracil (5-FU) by the enzyme cytosine deaminase (Fca1), which is present in fungal cells but absent in human cells, conferring selectivity to the drug. Subsequently, 5-FU is metabolized into 5-fluorouridine monophosphate (FUMP) by uracil phosphoribosyltransferase (UPRT), and incorporated into fungal RNA in place of uracil. This disrupts protein synthesis and inhibits thymidine synthesis, leading to impaired DNA replication. The cumulative effect results in toxicity that halts fungal cell proliferation and repair mechanisms [5,18].

However, resistance to 5-FC is commonly reported, particularly in *Candida* species, and is often associated with defects in the enzymatic conversion steps that are crucial to the drug’s efficacy. Documented resistance mechanisms include mutations in the *FCY1* gene, which encodes Fca1, impairing the conversion of 5-FC to 5-FU [4,18]. Fungal cells may also lose or modify the UPRT enzyme, encoded by FUR1, which blocks the conversion of 5-FU to FUMP. In addition, *Candida* species can develop mechanisms to counteract RNA and DNA damage through the overexpression of repair enzymes such as thymidylate synthase and the activation of stress response pathways, including the Hog1 MAPK pathway [5,36].

Given these resistance concerns, 5-FC is typically reserved for combination therapies, aiming to overcome resistance, reduce toxicity, or enhance antifungal efficacy through synergistic effects. However, its use requires careful evaluation of pharmacological interactions and therapeutic drug monitoring to ensure safety and effectiveness [37].

In summary, the increasing prevalence of antifungal resistance in *Candida* spp. underscores the need for a comprehensive understanding of the mechanisms involved in drug resistance across different classes of antifungal agents. Azoles, polyenes, and echinocandins, while effective, face significant challenges due to genetic mutations, efflux pump overexpression, and biofilm formation in resistant strains. These mechanisms not only limit the efficacy of current treatments but also emphasize the necessity for continued research into novel therapeutic strategies. Exploring alternative antifungal agents, understanding regional variations in resistance patterns, and investigating synergistic drug combinations remain critical to overcoming the global challenge of fungal infections.

## 4. Medicinal Plants

The medicinal use of plants dates back thousands of years, with China and India among the most prominent countries with histories of such use in therapeutic practices. Plants have been utilized in various forms, including teas, compresses, baths, and extracts. With the growing availability of information and discoveries, both the pharmaceutical and food industries have shown increasing interest in exploring the multiple applications of medicinal plants. This renewed attention to traditional practices highlights their valuable potential for developing new treatments and innovative products [9,38].

**Figure 3:**
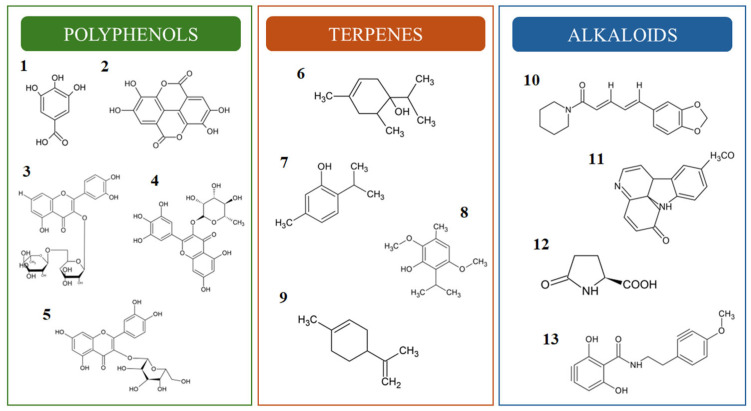
Chemical structures of the most prevalent compounds with anti-*Candida* activity. Polyphenols: gallic acid (**1**), ellagic acid (**2**), rutin (**3**), myricitrin (**4**) and hyperoside (**5**). Terpenes: 4-terpineol (**6**), thymol (**7**), esoespitanol (**8**) and limonene (**9**). Alkaloids: piperin (**10**), methylamine (**11**), acid pyroglutamic (**12**) and riparin III (**13**).

Medicinal plants produce secondary metabolites that play a crucial role in their defense mechanisms and biological functions. These chemical components, characterized by complex structures, exert diverse mechanisms of action in biological activities [39]. Their properties include anti-inflammatory, antimicrobial, antioxidant, anticancer, neuroprotective, and immunostimulant activities. Phytochemical studies have identified polyphenols, terpenes, and alkaloids (Figure 3) as key compounds in plant species exhibiting these activities. This has led to extensive research into their therapeutic potential and mechanisms of action [40,41,42].

The growing interest in natural alternative medicines with antifungal activity stems from the limitations of conventional antifungal agents. These synthetic drugs often present undesirable side effects and are increasingly challenged by the problem of acquired resistance [7,43]. Plant species, being natural sources, offer a promising alternative due to their chemical diversity, producing bioactive compounds effective against a broad range of pathogens [44,45].

Plant-derived compounds have gained increasing attention as promising alternatives for managing fungal infections, particularly those caused by drug-resistant *Candida* species. One of the most compelling advantages of these compounds lies in their multifactorial mechanisms of action, which make it more difficult for fungal cells to develop resistance [6,7]. Unlike synthetic antifungals, which often act on single molecular targets, plant metabolites can simultaneously affect various cellular pathways, including membrane integrity, metabolic function, and virulence gene expression. These properties not only enhance their fungicidal or fungistatic activity but also reduce the likelihood of resistance development. Furthermore, many phytocompounds exhibit synergistic effects when combined with conventional antifungals, allowing dose reductions and minimizing side effects. Their low cytotoxicity to human cells also contributes to a more favorable safety profile [6,46,47,48,49,50].

In this context, the selection of studies included in Table 2 was based on stringent criteria to ensure data quality, relevance, and reproducibility. Specifically, we included only studies that (1) clearly identified the chemical constituents responsible for the antifungal activity, and (2) provided minimum inhibitory concentration (MIC) values obtained through well-defined and standardized susceptibility testing against *Candida* spp. Based on this, Table 2 presents the main compounds involved in anti-*Candida* activity studied in recent years, with a focus on different classes of secondary metabolites and their diverse antifungal mechanisms.

Notably, bioactive compounds within the same chemical group can exhibit varying antifungal potentials, as evidenced by comparative results of MIC values, considering both fungistatic and fungicidal effects. These variations reflect structural properties as well as interaction with fungal targets.

### 4.1. Polyphenols Activity

Polyphenol compounds are widely recognized for their biological activities and are abundant in various plant species. Numerous studies highlight their primary antioxidant function, which protects organisms from oxidative stress, both internal and external. Flavonoids consist of the most abundant phenolic compounds, with three rings, and structural variations arising from substitutions in the heterocyclic pyran ring (C), which connects two aromatic rings (A and B). These compounds are classified into several subclasses, including flavonols, flavanones, flavones, flavanols, anthocyanidins, isoflavones, and chalcones [80,81].

Flavonoids are synthesized through the shikimate and acetate pathway, an essential metabolic route that produces aromatic compounds from simple precursors. This process begins with the conversion of shikimic acid into cinnamic acid, a crucial step in flavonoid biosynthesis. The diversity of flavonoids results from catalytic processes that introduce modifications such as hydroxylation, methylation, acetylation, or glycosylation, generating a wide array of compounds [50,81].

The chemical diversity of flavonoids underpins their varied biological activities, with distinct mechanisms of action arising from modifications within their subclasses [49]. Among their activities, antifungal effects have been extensively studied, with targets including fungal cell membranes and biofilm formation. Research indicates that flavonoids may exhibit multiple mechanisms of action, such as binding to ergosterol, inhibiting biofilm development, and inducing oxidative stress, collectively suppressing fungal proliferation. Furthermore, the synergistic potential of flavonoids with existing antifungal drugs has garnered growing interest, as many conventional agents face resistance from fungal strains. These studies suggest that combining flavonoids with antifungals could enhance their efficacy, offering new therapeutic alternatives against fungal infections [10,82].

Tenorio et al. [65] evaluated the antifungal activity of *Eugenia uniflora* leaf extracts and enriched fractions against three *Candida* species. The dry extract exhibited MICs of 250, 125, and 31.2 µg/mL against *C. albicans*, *C. glabrata*, and *C. auris*, respectively. Similarly, the ethyl acetate fraction displayed identical MIC values, whereas the hexane fraction was effective only against *C. auris* with a MIC of 62.5 µg/mL. The flavonoid-rich fraction showed significant activity against *C. albicans* (125 µg/mL) and *C. glabrata* (62.5 µg/mL). The final fraction (LF) achieved an MIC of 125 µg/mL for all three strains tested. Additionally, the ellagic acid-rich fraction demonstrated significant activity against *C. glabrata* and *C. auris*, with MICs of 62.5 and 125 µg/mL, respectively. These results highlight the critical role of flavonoids in the antifungal activity of *E. uniflora* extracts, corroborating findings from other studies on the bioactive potential of these compounds.

The antifungal activity of the ethanolic extract of *Annona muricata*, with markers such as rutin, was evaluated against *C. albicans* strains. The study demonstrated that the extract acts on the fungal membrane and cell wall, with an eightfold increase in MIC in the presence of ergosterol and sorbitol. Additionally, the study assessed metabolic activity and membrane integrity, showing reduced *C. albicans* activity in the presence of the extract, with results comparable to or better than the antifungal nystatin [36]. Flavonoids compounds were reported in [83] with anti-*Candida albicans* activity. Synergism assays were performed using the aqueous and ethanolic extracts of *Bauhinia holophylla*, combining both extracts with fluconazole and the ethanolic extract with chlorhexidine gluconate. These combinations exhibited synergistic effects and inhibited germ tube formation compared to the individual actions of the components.

da Costa Cordeiro et al. [55] reported the antifungal effect of *Spondias tuberosa* extract against *C. albicans*, with MIC values of 2.0 mg/mL, and against *C. glabrata*, with MIC values of 0.078 mg/mL. The extract showed greater efficacy against *C. glabrata*. Metabolic activity was also assessed, revealing that the extract induced cellular damage in *C. glabrata*. These findings highlight the critical role flavonoids play in reducing the virulence of *Candida* spp.

Meccatti et al. [71] demonstrated that glycolic extracts from *Punica granatum*, *Rosmarinus officinalis*, *Curcuma longa*, and *Rosa centifolia* were active against fluconazole-resistant strains such as *Candida krusei*. The antifungal effects were attributed to the presence of hydrolyzable tannins, quercetin derivatives, *p*-coumaric acid, and curcumin, compounds known to act through disruption of membrane integrity, inhibition of ergosterol biosynthesis, suppression of biofilm formation, and induction of oxidative stress, mechanisms that not only kill fungal cells but also interfere with their capacity to persist and invade host tissues.

Expanding on this, Mendonca et al. [72] reported that galloyl-HHDP-glucose (G-HHDP-G), a polyphenol isolated from *P. granatum*, demonstrated significant synergistic antifungal activity when combined with fluconazole, especially against *C. albicans* and *C. glabrata*. This synergy led to a marked reduction in MIC values for both the polyphenol and the antifungal drug, indicating enhanced efficacy and the possibility of dose reduction. In addition, G-HHDP-G inhibited biofilm formation and disrupted mature biofilms, further supporting the role of polyphenols in targeting fungal virulence traits. Together, these studies underscore that the antifungal activity of polyphenols extends beyond direct fungicidal effects and includes the modulation of key pathogenic processes, making them highly relevant in the context of antifungal resistance and therapeutic innovation.

Flavonoids such as avicularin, baicalein, and quercetin have demonstrated significant antifungal activity, particularly against fluconazole-resistant *Candida* strains, and are increasingly recognized for their ability to impair virulence rather than solely exert fungicidal effects. Avicularin, a quercetin glycoside, significantly inhibited *C. albicans* adhesion to oral epithelial cells and suppressed hyphal growth, two critical steps for tissue invasion and biofilm formation [62]. These effects were attributed to avicularin’s capacity to interfere with the fungal cytoskeleton and cell wall remodeling, as well as its modulation of surface adhesin expression, thereby reducing colonization and persistence in the host environment. In parallel, baicalein and quercetin, when used alone or in combination, exhibited a synergistic antifungal effect characterized by disruption of membrane integrity, increased permeability, and leakage of intracellular proteins, which together compromise fungal cell viability. Importantly, they also act at the transcriptional level by downregulating virulence-associated genes such as *ALS1*, *ALS3*, *HWP1*, *EFG1*, *SAP4*, and *CPH1*, many of which are directly involved in hyphal formation, biofilm maturation, and immune evasion [68]. This combination of structural and molecular interference suggests a multi-targeted approach that reduces the potential for resistance development.

Additionally, ethanolic and hexane extracts from *Capsicum chinense*, rich in phenolic acids (e.g., gallic, ferulic, and *p*-coumaric acids), quercetin glycosides, and capsaicinoids (mainly capsaicin), have shown broad-spectrum antifungal and antivirulence effects [73]. These compounds act by inhibiting the synthesis and function of fungal enzymes, suppressing hemolysin production, a key factor for host cell lysis and iron acquisition, and preventing biofilm formation, particularly in *C. glabrata* and *C. tropicalis*. Furthermore, their lipophilic nature allows them to integrate into fungal membranes, disrupting membrane potential and increasing oxidative stress. Such mechanisms not only impair fungal growth but also sensitize cells to conventional antifungal agents, suggesting their potential as adjuvants in antifungal therapy.

Retore et al. [78] investigated the ethanolic extract of *Caryocar brasiliense* against various *Candida* species, with particular emphasis on fluconazole-resistant *Candida auris* strains. The study also characterized the polyphenolic constituents of the extract. Antifungal activity was observed against *C. albicans*, *C. glabrata*, *C. tropicalis*, and resistant isolates of *C. auris*, with minimum inhibitory concentrations (MICs) ranging from 32 to >265 µg/mL. Notably, a synergistic effect between the extract and fluconazole was demonstrated against *C. auris*. The proposed mechanisms of action include inhibition of fungal cell growth, damage to the cell wall, and disruption of plasma membrane integrity, highlighting the extract as a promising candidate for the development of combination therapies.

Tonningianin A, an ellagitannin isolated from the *Terminalia* genus, was recently investigated for its antifungal potential against *Candida* species. The study yielded particularly promising results, showing antifungal activity significantly superior to that of fluconazole, one of the most widely used azole antifungals in clinical practice. Tonningianin A exhibited MIC_90_ values ranging from 2 to 8 µg/mL, whereas fluconazole showed MICs exceeding 64 µg/mL against the same strains. Furthermore, the compound demonstrated potent inhibition of *C. albicans* biofilm formation as well as disruption of mature biofilms. This study not only validates the antifungal potential of tonningianin A but also reinforces the importance of continued research into novel plant-derived metabolites with promising applications in the treatment of fungal infections, especially for strains that exhibit resistance to fluconazole. The observed reduction in minimum inhibitory concentration upon addition of tonningianin A suggests its ability to circumvent fluconazole resistance mechanisms, indicating a potential synergistic effect that could help reverse resistance. These findings are particularly relevant in the ongoing search for new therapeutic alternatives against drug-resistant *Candida* strains [84].

Taken together, these findings reinforce the role of flavonoids and related phenolic compounds as multifaceted agents capable of targeting fungal viability, morphology, and virulence, offering a strategic advantage in combating resistant *Candida* infections.

### 4.2. Terpene Activity

Terpenes are a class of phytochemicals predominantly found in plants, where they play critical roles in signaling, thermoprotection, and the release of pigments and aromas. Many terpenes possess toxic properties, functioning as chemical defenses against microorganisms and herbivores [85]. Additionally, these compounds exhibit a wide range of biological activities, including antidiabetic, anticancer, anti-inflammatory, and antimicrobial effects. Terpenes are synthesized through the mevalonic acid (MVA) and methylerythritol phosphate (MEP) pathways, resulting in the production of isopentenyl diphosphate (IPP) and dimethylallyl diphosphate (DMAPP), the precursors of various terpenes [86,87].

The isoprenoid precursors, composed of five carbon atoms, are fundamental to the diverse bioactive properties of terpenes. Structural functionalization and bioactivity variations arise from the action of terpene synthase (TPS) enzymes and cytochrome P450 monooxygenases. These enzymes drive the formation of specific compounds within each terpene class, including monoterpenes, sesquiterpenes, diterpenes, and triterpenes, distinguished by the number of carbon atoms in their chemical structures [87,88,89].

Terpenes, including monoterpenes, sesquiterpenes, and triterpenes, represent a diverse class of natural products with well-documented antifungal properties, particularly due to their lipophilic nature, which facilitates interaction with fungal membranes. These compounds often exert their effects by disrupting membrane integrity, interfering with mitochondrial function, and modulating oxidative pathways, mechanisms that are particularly valuable in overcoming drug resistance in *Candida* species [90].

In this context, Zahara et al. [91] isolated linoleic acid and dehydroabietic acid from *Bidens bipinnata*, both of which demonstrated potent antifungal activity against *C. albicans* and *C. krusei*. Linoleic acid, a polyunsaturated fatty acid, integrates into the fungal membrane bilayer, increasing fluidity and permeability, ultimately leading to cell lysis. Dehydroabietic acid, a diterpenoid resin acid, is believed to impair oxidative phosphorylation by targeting mitochondrial respiration, thereby reducing fungal energy production and viability. The study also highlighted a synergistic interaction between these compounds, which may potentiate their therapeutic effect and reduce the required dose for antifungal efficacy.

In another study, Soliman et al. [74] identified dehydrocostus lactone, a sesquiterpene lactone from *Saussurea costus*, as the main active constituent responsible for marked antifungal activity. This compound induced significant morphological alterations in *Candida* cells, including membrane rupture, cytoplasmic leakage, and surface wrinkling, as observed by scanning electron microscopy (SEM). Its potent activity against fluconazole-resistant strains and stability when applied to medical-grade cotton suggests it may be a strong candidate for topical antifungal formulations.

Moreover, triterpenoids such as 3-oxo-friedelan-20α-oic acid, betulinic acid, and oleanolic acid, isolated from *Solanum torvum*, have shown broad-spectrum antifungal action. These compounds displayed MICs as low as 0.016 mg/mL and significantly enhanced the efficacy of fluconazole, reducing its MIC by up to 16-fold in resistant *C. albicans* isolates [68]. Their mode of action includes disruption of the fungal cell wall and membrane, as well as inhibition of biofilm formation in a concentration-dependent manner. The biofilm-inhibitory property is particularly relevant given that biofilms are a major contributor to antifungal resistance and persistence in host tissues.

Overall, these findings demonstrate that terpenes, through membrane perturbation, mitochondrial targeting, and inhibition of virulence factors such as biofilms, exhibit a multifactorial antifungal profile. Their ability to act synergistically with conventional antifungals and their suitability for both systemic and topical applications position them as promising candidates for integration into next-generation antifungal therapies aimed at combatting resistant *Candida* infections.

Essential oils, rich in terpenes, also are known for their diverse biological activities, including antifungal properties. Their antifungal action is primarily mediated through the disruption of fungal cell membranes, leading to inhibited growth and cell death [92,93]. The interactions among the various compounds in essential oils can further enhance their biological activities. Given the increasing resistance of fungi to conventional antifungals, the development of studies focusing on plant species containing effective natural compounds against diverse fungal pathogens is critical [94,95]. This approach is particularly relevant in addressing current challenges in antifungal therapy.

Zahabib et al. [96] and colleagues evaluated the methanolic crude extract and various fractions of *Salvia rhytidea* Benth leaves, with nystatin as a control. The crude extract exhibited superior antifungal activity with MIC values of 15.625 µg/mL for *C. albicans*, 31.25 µg/mL for *C. tropicalis*, and 62.5 µg/mL for *C. parapsilosis*, compared to nystatin (64, 32, and 64 µg/mL, respectively). Regarding fungicidal potential, the extract outperformed nystatin only for *C. albicans* (31.25 µg/mL vs. 128 µg/mL). Although no phytochemical analysis was conducted, this plant species is known to contain various terpenoid compounds that are likely to contribute to its antifungal activity.

The antivirulence capacity of *Origanum vulgare* essential oil (Ov-EO) was tested by evaluating its biofilm inhibition, effects on planktonic cells, and synergistic interactions with fluconazole and nystatin. The findings demonstrated activity against two strains of *C. albicans* (ATCC 90029 and ATCC 10231), *C. krusei*, and *C. dubliniensis*, with the most effective action observed against ATCC 90029. Virulence factor inhibition revealed that Ov-EO had a stronger effect on filament inhibition and cellular adhesion compared to the antifungal agents tested. Furthermore, Ov-EO exhibited several synergistic interactions with fluconazole and nystatin [52].

Essential oils from *O. glandulosum* (Og-EO), *A. verticillata* (Av-EO), and *S. satureioides* (Ss-EO), containing compounds similar to those looked at in the previous study, also showed antibiofilm activity against *C. albicans* and *C. glabrata* isolates. However, synergistic interactions with amphotericin B were only observed for Og-EO in one *C. albicans* strain [92].

Malveira et al. [59] studied the essential oil of *Croton blanchetianus* (Cb-EO) and evaluated its mechanisms of action against *C. albicans* and *C. parapsilosis*. Membrane damage in planktonic cells was more pronounced in *C. parapsilosis*. Additionally, Cb-EO induced membrane permeabilization in biofilms of both species, with greater efficacy observed in *C. albicans* biofilms.

In the evaluation of antimicrobial activity against resistant microorganisms, the essential oil of *Juniperus thurifera* L. demonstrated effective inhibitory action against most of the tested strains, including the bacterium *Pseudomonas aeruginosa* and the fungi *Candida albicans* and *Fusarium oxysporum*. The selected strains were resistant to conventional antimicrobials such as streptomycin and erythromycin (for bacteria), and fluconazole (for fungi), highlighting the potent effect of the essential oil, with MICs ranging from 0.0475 to 0.095 µg/mL. This promising activity may be associated with the presence of major constituents identified in the oil, including α-thujene, elemol, and muurolol, which were present at the highest concentrations. These results support the potential of *J. thurifera* essential oil as a therapeutic alternative for combating infections caused by multidrug-resistant pathogens [76].

These studies highlight how terpenoid compounds can disrupt fungal cell integrity, impairing functionality and inducing apoptosis.

### 4.3. Alkaloid Activity

Alkaloids are secondary metabolites widely distributed in plants, characterized by the presence of nitrogen atoms in their structures. These compounds exhibit diverse biological activities, including antimicrobial, anticancer, anti-inflammatory, analgesic, and antioxidant properties. Additionally, research highlights their prominent bioactive effects, particularly of a psychoactive nature. Alkaloids are classified based on the number and arrangement of nitrogen groups in their structures [97,98,99].

These compounds are derived from aromatic amino acids and are biosynthesized mainly through the shikimate pathway. This pathway leads to the formation of amino acids such as tryptophan, tyrosine, ornithine, and lysine, which give rise to indole, isoquinoline, tropane, and piperidine alkaloids, respectively [97,100]. Among the most notable alkaloids with bioactive properties are atropine, morphine, codeine, nicotine, and quinine [101].

Due to their structural diversity, alkaloids exhibit various antifungal mechanisms of action, targeting different active sites across fungal species. Additionally, they are known for their low toxicity, making them strong candidates for the development of new antifungal agents. Given the increasing resistance of pathogenic fungi such as *Candida* spp., alkaloids have shown promise as both inhibitors and fungicidal agents [102,103].

A study by da Silva et al. [63] on *Aniba riparia* isolated Riparin compounds (I, II, III, and IV) and evaluated their antifungal activity compared to fluconazole. Riparin III exhibited activity against *C. albicans*, *C. tropicalis*, and *C. krusei* with MIC values of 256, 128, and 256 µg/mL, respectively. When compared to fluconazole, Riparin III demonstrated superior activity against *C. albicans* (>1024 µg/mL) and *C. tropicalis* (1024 µg/mL). The other three Riparin isolates showed better antifungal activity against *C. albicans* (1024 µg/mL) compared to fluconazole. However, for *C. krusei*, fluconazole exhibited superior efficacy with an MIC of 128 µg/mL. Combination assays between fluconazole and Riparin isolates revealed synergistic effects, with IC_50_ MICs of 8 µg/mL (Riparin III + fluconazole) and 16 µg/mL (Riparin II + fluconazole) for *C. albicans* and *C. tropicalis*, respectively.

Similarly, Bravo-Chaucanés et al. [61] evaluated the susceptibility of ethanolic extract and various fractions of *Piper nigrum* against *C. albicans* and *C. auris*. The study investigated biofilm formation inhibition, morphological transition, and metabolic activity. The extract demonstrated superior effects for both strains compared to the fractions. Regarding biofilm formation, the extract showed higher inhibition in *C. auris* strains. In terms of yeast-to-hyphae transition in *C. albicans*, the extract effectively suppressed this process. Additionally, the secretion of hydrolases, which are key virulence factors, was reduced in the presence of ethanolic extract.

A compound isolated from *Aerva lanata*, methylervine, was explored for its anticandidal potential and possible mechanisms of action. Various *C. albicans* strains were tested, with significant growth inhibition observed for *C. albicans* ATCC 90028 (MIC of 32 µg/mL) and ATCC 90029 (MIC of 16 µg/mL). Virulence factors such as biofilm formation inhibition, hyphal formation suppression, spore and cell morphological damage, and fungal DNA damage were also observed, demonstrating that the compound exerts multiple mechanisms of action [104].

As demonstrated in the study by Duan et al. [66], the extract of *Pachysandra axillaris* Franch and its isolated alkaloids exhibited antifungal activity superior to nystatin against fluconazole-resistant *Candida albicans* strains. The study elucidated the mechanisms of action of the alkaloid pachysamine M, which included modulation of ergosterol biosynthesis by downregulating the expression of key regulatory enzymes such as those from the *ERG* gene family, thereby disrupting cell membrane homeostasis. Additionally, structural alterations to the membrane were observed, including increased permeability and reduced fluidity. The treatment also led to the accumulation of intracellular reactive oxygen species (ROS), which correlated with mitochondrial dysfunction and the induction of apoptosis.

Alkaloids such as solasonine and solamargine, along with steroidal lactones derived from plants of the *Solanaceae* family, have demonstrated notable antifungal activity against *Candida* species, including strains resistant to conventional antifungal agents. These compounds have also shown efficacy against other clinically relevant fungal pathogens, such as *Sporothrix brasiliensis*, further highlighting their broad-spectrum potential. MIC values for solasonine and solamargine ranged from 0.125 to 1 mg/mL, with both exhibiting fungistatic and fungicidal effects. The antifungal mechanisms are primarily associated with the disruption of fungal cell wall integrity and membrane permeability, mechanisms that are likely shared across different fungal genera. These findings reinforce the therapeutic relevance of *Solanaceae*-derived alkaloids as versatile antifungal agents capable of targeting both drug-resistant *Candida* strains and other pathogenic fungi through conserved molecular pathways [105,106].

In the study by Shen et al. [77], the ethanolic extract and fractions of *Sarcococca hookeriana* demonstrated significant antifungal activity against *Candida albicans* strains resistant to fluconazole. Phytochemical analysis identified sarcovagine D as the main bioactive compound, particularly enriched in the SHE80 fraction, which exhibited a minimum inhibitory concentration (MIC) of 16 µg/mL, clinically relevant when compared to standard antifungal agents. Moreover, the study employed metabolomic profiling coupled with multivariate statistical analysis to correlate chemical composition with antifungal effects. The findings suggest that the active fraction acts by inhibiting biofilm formation and hyphal transition, which are critical virulence factors of *C. albicans*. Additionally, the extract contributed to wound healing in a murine model of cutaneous candidiasis.

Chelerythrine, a benzo[*c*]phenanthridine alkaloid isolated from *Macleaya cordata*, has shown potent activity against fluconazole-resistant *Candida albicans*. Chelerythrine inhibited both biofilm formation and viability of mature biofilms, critical determinants of antifungal resistance and persistence [70]. Mechanistically, its antifungal action involves disruption of cell surface hydrophobicity, inhibition of the cAMP–PKA–Efg1 signaling pathway—which governs hyphal development and biofilm architecture—and induction of reactive oxygen species (ROS) accumulation, leading to oxidative damage and cell death. The ability to modulate both structural and regulatory elements of virulence makes chelerythrine a notable candidate for targeting recalcitrant fungal infections.

Similarly, arborinine and graveoline, two alkaloids isolated from *Ruta angustifolia*, demonstrated strong fungicidal activity by targeting isocitrate lyase (ICL1), an enzyme integral to the glyoxylate cycle, a metabolic pathway that supports fungal survival in nutrient-limited environments such as inside macrophages or biofilms [69]. By suppressing *ICL1* gene and protein expression, these compounds effectively impair the metabolic flexibility of *Candida albicans*, reducing its ability to adapt, persist, and evade immune responses, thus weakening its pathogenicity.

Further highlighting the antifungal potential of alkaloids, moscatin, a phenanthrene alkaloid from *Dendrobium nobile*, demonstrated broad-spectrum activity against fluconazole-resistant *Candida albicans* isolates [75]. Moscatin significantly downregulated genes involved in ergosterol biosynthesis (*ERG1*, *ERG11*), hyphal development (*HWP1*, *SAP3*), and adhesion (*ALS2*, *ALS4*), thereby attenuating key virulence factors. Notably, in in vivo murine models, moscatin not only reduced fungal burden and improved survival rates but also contributed to the restoration of gut microbial diversity disrupted by infection, an attribute that supports its potential as both an antifungal and microbiota-preserving therapeutic.

Taken together, the accumulated evidence underscores that polyphenols, terpenes, and alkaloids from medicinal plants exhibit complementary and often synergistic antifungal mechanisms. These include membrane disruption, metabolic inhibition, suppression of virulence gene expression, inhibition of biofilm formation, and synergy with conventional antifungals, positioning them as powerful tools against emerging antifungal resistance. As comprehensively reviewed by Huang et al. [90], many of these compounds act on targets not affected by conventional drugs, and their biosynthetic pathways are increasingly being elucidated. This knowledge enables the use of synthetic biology and metabolic engineering approaches to optimize compound production and enhance pharmacological profiles, paving the way for the development of next-generation antifungal therapies based on plant-derived bioactives.

## 5. Innovative Technological Approaches for Assessing and Enhancing Antifungal Efficacy

The technological advancements to enhance the availability of natural products with diverse biological activities have become increasingly extensive and crucial for various reasons. The need for different health applications and the investigation of resistance mechanisms contribute to innovations essential for combating fungal infections [39]. Key factors driving this progress include the rise in resistance to synthetic antifungals, the therapeutic efficacy of multiple bioactive compounds, the diversity of therapeutic alternatives, less invasive treatments, and reduced reliance on synthetic drugs [107,108,109].

Studies on mechanisms of action are explored using various techniques, such as microscopy, with key methods including scanning electron microscopy (SEM), transmission electron microscopy (TEM), and fluorescence assays. These techniques allow the visualization of cellular damage, such as changes in membranes and cell walls, caused by treatment with plant-derived compounds [36,59,60,64,104]. Another widely used approach involves molecular assays, such as reverse transcription polymerase chain reaction (RT-PCR) and transcriptomic analyses, including RNA sequencing (RNA-Seq). These methods enable the evaluation of gene expressions associated with cellular composition, molecular functions, and biological processes. Additionally, they allow for the differentiation of gene and protein regulation, facilitating the identification of cellular types involved in responses to fungal infections [110,111,112,113].

Proteomics-, genomics-, and metabolomics-based approaches have revolutionized the identification of new bioactive compounds and the elucidation of their antifungal mechanisms of action. These technologies enable integrated, large-scale analyses of the molecular components of microorganisms, providing critical insights for the development of more effective therapeutic strategies [114]. Genomic and proteomic tools can be used to compare susceptible and resistant fungal strains, allowing for the identification of key proteins and alterations in metabolic pathways. Meanwhile, metabolomics supports the comprehensive characterization of bioactive plant extracts as well as the metabolic profiling of fungal cells. When used in combination, these approaches can optimize the identification of potential antifungal compounds and help predict interactions between plant-derived substances and fungal targets [115].

Molecular modeling techniques, such as molecular docking, are valuable for investigating the potential binding targets of plant-derived compounds. These bioactive markers interact with proteins responsible for fungal cell functionality, exhibiting specific binding capacities that reduce metabolic activity, cause cellular damage, and inhibit cell growth [31,54]. This computational approach has become an indispensable tool in the design and detailed analysis of interactions between candidate compounds and their biological targets. These interactions are primarily evaluated through binding energy, which serves as a critical quantitative parameter to predict molecular affinity and specificity. By simulating molecular docking and assessing the strength of these interactions, computational models help guide the selection of the most promising bioactive compounds for further experimental validation [116].

Regarding the delivery of active compounds, one of the most significant recent methodological advances against fungal infections is the development of antifungal films, particularly those incorporating bioactive plant extracts, either alone or in combination with conventional antifungal agents [117,118]. Among emerging technologies, 3D printing has gained increasing attention for its capacity to produce customized films with innovative applications in surface coating and controlled drug release. These polymer-based delivery systems represent a promising alternative for localized antifungal treatment, particularly in scenarios such as wound healing, catheter protection, and implantable medical devices. However, future studies must address key challenges, including scalability of production, as well as the chemical and physical stability of natural compounds when subjected to formulation processes and long-term storage [119,120].

Pharmaceutical technologies have also been developed to address the increasing resistance mechanisms. Alternative approaches include dispersed systems, nanostructured systems, and biosensors. These drug delivery systems represent a significant advancement in the administration of antifungal agents, overcoming many of the limitations associated with conventional formulations. Nanocarriers function as intelligent delivery vehicles, capable of protecting the active compound from premature degradation while enhancing its distribution to target tissues [121]. This molecular protection is particularly valuable for compounds with low solubility or chemical instability, as it improves bioavailability and therapeutic effectiveness. In parallel, biosensors emerge as innovative tools for early detection of fungal infections. By combining biological recognition elements with physicochemical transducers, these systems enable rapid and sensitive identification of specific biomarkers associated with infectious processes [122]. These devices enable the encapsulation of constituents such as essential oils, extracts, fractions, and isolated compounds, improving stability and providing efficient release profiles for infection treatment [108,116].

Another promising tool is artificial intelligence (AI), which is rapidly emerging as a valuable resource for improving both diagnostic strategies and the discovery of novel therapeutic agents. Through data-driven approaches, AI enables morphological pattern recognition, virtual screening, and the prioritization of natural products with potential antifungal activity. These approaches can accelerate the identification of lead compounds effective against *Candida* infections, thereby complementing traditional methods and enhancing the efficiency of natural product-based drug discovery [123,124]. Research efforts toward new technological applications contribute significantly to providing effective treatment options for infections caused by *Candida* spp.

## 6. Conclusions

This study highlighted the diverse resistance mechanisms in *Candida* spp., including specific targets and mutations contributing to antifungal inefficacy. In response to this, it emphasized the importance of research on medicinal plants and their rich phytochemical compositions, which encompass a wide range of bioactive activities, including antifungal properties. Additionally, various chemical compounds such as polyphenols, terpenes, and alkaloids demonstrated significant antifungal potential, often at low concentrations, exhibiting both fungistatic and fungicidal effects. Many of these studies have elucidated potential mechanisms of action for secondary metabolites, providing valuable insights into fungal cell targets. The challenges in discovering new antifungal agents derived from natural sources remain crucial to addressing the growing issue of antifungal resistance. Advancing this field will be fundamental in mitigating the impact of resistance in fungal infections and expanding the arsenal of effective treatment options.

## Figures and Tables

**Table 1:** Resistance mechanisms identified in *Candida* spp. strains for clinically used antifungal agents.

Classes of Antifungal Agents	Resistance Genes	References
Azoles Fluconazole	*CDR1*, *CDR2* (overexpression of efflux pumps) *MDR1* (drug transporter) *TAC1* (gain-of-function mutations/transcription factors) *ERG3* and *ERG11* (genes encoding lanosterol 14α-demethylase)	[21,22,23]
Polyenes Amphotericin B	*ERG3*, *ERG5*, *ERG6*, *ERG11* (target genes—ergosterol biosynthesis) *FKS1*	[12,24,25]
Echinocandins Caspofungin	*FKS1* and *FKS2* (genes encoding β-1,3-D-glucan synthase) *ERG3* (gene encoding sterol Δ5,6-desaturase)	[24,26,27,28]
Pyrimidine 5-Fluorocytosine	*FUMP* (blocking formation) *FCY1* and *FUR1* (decreased uranil phosphoribosyltransferase-UPRTase activity)	[4,5,11,18]

**Table 2:** Bioactive Compounds from Plant Species, Mechanisms of Action, and Antifungal Potential Against *Candida* spp. Strains.

Plant/Derivative	Compounds/Identification Method	Mechanism of Action	*Candida* Species	MIC (μg/mL)	References
*Fridericia chica*/extract	Scutellarein-O-glucuronide and 5-methyl-scutellarein-O-glucuronide/UPLC-ESI-MS	Morphogenesis Inhibition	*C. albicans* (ATCC 10231) ^AR^ *C. albicans* (ATCC 18804) *C. albicans* (ATCC 2091) *C. albicans* (SC 5314) *C. albicans* Isolates *C. krusei* (ATCC 34135) ^FR+RI^	512 512 512 512 256 to 1.024 512	[51]
*Annona muricata*/extract	Rutin, kaempferol-3O-rutinoside, xylopine, and caffeic acid/UFLC-QTOF-MS	Damage to the Fungal Membrane and Envelope	*C. albicans* (ATCC 10231) ^AR^	1000	[36]
*Origanum vulgare*/Essential oil	Thymol, 4-terpineol, and γ-terpinene/GC-MS	Anti-Adhesion and Morphogenesis Inhibition	*C. albicans* (ATCC 90029) *C. albicans* (ATCC 10231) ^AR^ *C. krusei* (ATCC 6258) ^FR+RI^ *C. dubliniensis* (CD36)	0.01 0.97 5.33 2.61	[52]
*Erythrina senegalensis*/extract	Neobavaisoflavone, alpinumisoflavone, cristacarpine, and 6,8-diprenylgenistein/UPLC-ESI-QTOF-MS/MS	Multiple	*C. albicans* (ATCC 10231) ^AR^ *C. albicans* (SC5314) ^FR^ *C. glabrata* (ATCC 2001) ^AR^	15.63 31.25 7.81	[53]
*Eugenia uniflora*/extract	Gallic acid and myricitrin/HPLC-DAD	Binding to α-14-Sterol Reductase and 1,3-β-Glucan Synthase	*C. albicans* (ATCC 90028) *C. dubliniesis* (CBS 7987) *C. tropicalis* (ATCC 13803) *C. parapsilosis* (ATCC 22019) *C. glabrata* (ATCC 2001) ^AR^	312.5 312.5 625 312.5 625	[54]
*Spondias tuberosa*/extract	Hyperoside and gallic acid/HPLC-DAD and NMR	High Levels of Mitochondrial Superoxide Anion, Hyperpolarization, and Lysosomal Membrane Damage	*C. albicans* Isolates *C. glabrata*	2000 78	[55]
*Callistemon citrinus*/extract	Tormentic acid/NMR	Membrane Damage	*C. albicans* (NCPF 3255) ^FR^	100	[56]
*Cinnamomum verum*/essential oil	Cinnamaldehyde, 1,8-cineole, and α-copaene/GC-MS	Unknown	*C. albicans* (ATCC 10231) ^AR^	62.5	[57]
*Brysonima gardneriana*/extract	Pyroglutamic acid, eucalyptol, and octanoic acid/GC-MS	Membrane Disruption and Oxidative Stress	*C. albicans* (ATCC 10231) ^AR^ *C. glabrata* (ATCC 90030) *C. krusei* (ATCC 6258) ^FR+RI^	125 125 250	[44]
*Myrtus communis*/extract and fraction	Terpenes/Total terpenoids content	Unknown	*C. albicans* (ATCC 76645) ^FR^ *C. albicans* resistant to nystatin	62.5 to 250 62.5 to 250	[58]
*Croton blanchetianus*/essential oil	Limonene, sabinene, terpinen-4-ol, borneol/GC-MS-MS	Plasma Membrane Damage and Oxidative Stress	*C. albicans* (ATCC 10231) ^AR^ *C. parapsilosis* (ATCC 22019)	50	[59]
*Oxandra xylopioides*/extract	Isoespintanol/GC-MS	Plasma Membrane Damage	*C. albicans* Isolates *C. auris* *C. glabrata* *C. tropicalis*	452.4 to 493 453.5 496 450.4 to 503.3	[60]
*Piper nigrum*/extract and fraction	Piperine/LC-MS QqQ	Cell Wall Lysis	*C. albicans *(SC5314) ^FR^ *C. albicans* (CAAL256) ^CI+FR^ *C. auris* (CAAU435) ^CI^ *C. auris* (CAAU537) ^CI+FR+ABR^	2048 1024 512 512	[61]
*Myrcia tomentosa*/extract and fraction	Avicularin/NMR	Anti-Adhesion and Morphological Alterations	*C. albicans* (ATCC 90028) *C. parapsilosis* (ATCC 22019)	32 16	[62]
*Aniba riparia*/ isolated	Riparin/NMR and ESI-MS	Efflux Pump Inhibition (*MDR1*, *CDR1*, and *CDR2* Genes)	*C. albicans* (ATCC 10231) ^AR^ *C. tropicalis* (ATCC 13803) *C. krusei* (ATCC 34135) ^FR+RI^	256 128 256	[63]
*Mitracarpus frigidus*/extract and fraction	Scopoletin/HPLC-PDA	Cell Wall, Plasma Membrane Sterols, and Efflux Pump Inhibition	*C. tropicalis* (ATCC 28707) ^ABR^	50	[64]
*Eugenia uniflora*/extract	Myricitrin, ellagic acid, and gallic acid/LC-ESI-HRMS/MS	Associated with Cell Wall Inhibition	*C. albicans* (ATCC 90028) *C. glabrata* (ATCC 2001) ^FR^ *C. auris* (CDC B11903) ^FR+ABR^	250 125 31.2	[65]
*Pachysandra axillaris*/extract	Sarcovagine D, Epipachysamine D, Pachysamine M/NMR/HR-ESI-MS	Inhibition of Ergosterol Biosynthesis (*ERG4*, *ERG7*, *ERG9*, *ERG1*, and *ERG24*), Disruption of Cell Membrane Homeostasis, and Accumulation of Oxidative Stress	*C. albicans* (SC5314) ^FR^	4	[66]
*Anacardium occidentale*/extract	Gallic acid, luteolin and agathisflavone/ UPLC-DAD/QTOF-MS	Increased Permeability of the Fungal Membrane	*C. albicans* (INCQS 40006) ^FR^ *C. krusei* (INCQS 40095) ^FR^ *C. tropicalis* (INCQS 40042) ^FR^	376.6 395.3 352.3	[67]
*Solanum torvum*/extract and fraction	3-oxo-friedelan-20α-oic acid, betulinic acid, oleanolic acid and sitosterol-3-β-D-glucopyranoside/NMR	Disruption of the Fungal cell Wall and Membrane	*C. albicans* Isolates ^FR^	250–2000	[68]
*Ruta angustifolia*/extract and fraction	Aborimine and graveoline/NMR/HPLC	Inhibition of the *ICL1* Gene	*C. albicans* (ATCC 10231) ^FR^	250–500	[69]
*Acleaya cordata*/extract	Chelerythrine/HPLC	Reducing the CSH, Inhibiting the cAMP Pathway, Increased cell Membrane Permeability and ROS Accumulation	*C. albicans* (SC5314) ^FR^ C. albicans (CA16) ^CI+FR^	2–128 8–128	[70]
*Rosmarinus officinalis*/extract	*p*-coumaric acid, chlorogenic acid and gallotannin/ HPLC-DAD	Increase Mitochondrial Depolarization, Production of Reactive Oxygen Species, and Causes DNA Fragmentation	*C. albicans* (ATCC 18804) *C. ddubliniensis* (ATCC MYA 646) *C. tropicalis* (ATCC 13803) *C. krusei* (ATCC 6258) ^FR+RI^	25,000–50,000	[71]
*Punica granatum*/extract	Gallotanin, quercentin/HPLC-DAD			50,000	
*Rosa centifolia*/extract	Derivative of quercetin, gallic acid, gallotannins, *p*-coumaric acid/HPLC-DAD			12,500–25,000	
*Curcuma longa*/extract	Curcumin/HPLC-DAD			25,000	
*Punica granatum/fraction*	Galloy-hexahydroxydiphenoyl-glucose/HPLC-DAD-ESI-IT/MS	Reducing *Candida* Phospholipase Production	*C. albicans* (ATCC 90028) *C. albicans* CAS ^CI^ *C. glabrata* (ATCC 2001) *C. glabrata* FJF ^CI^	>500 >500 125 31.25	[72]
*Capsicum chinense/extract*	Capsaicin and dihydrocapsaicin/LC-ESI-MS	Lyse of the Cell Wall, Inhibition of Hemolytic Activity	*Candida albicans* (ATCC 90028 and CI) *Candida glabrata* (ATCC 2001 and CI) ^FR^ *Candida krusei* (ATCC 6258 and CI) ^FR^ *Candida parapsilosis* (ATCC 22019 and CI) *Candida tropicalis* (ATCC 13803 and CI)	1500–3000 3000	[73]
*Saussurea costus*/extracts	Dehydrocostuslactone, 11,14,17-eicosatrienoic acid methyl ester/GC/MS	Cell Wall Damage	*C. albicans* (ATCC 10231) ^FR^ *C. tropicalis* (MH 445555) *C. parapsilosis* (MH 445556) *C. pseudotropicalis* (Biochemical ID) *C. guillimondii* (Biochemical ID) *Candida* Isolates	1000–3000 1000–4000 1000–3500 250–4500 1000–3000 200–4500	[74]
*Dendrobium nobile*	Moscatin	Modify Ergosterol Synthesis Pathway Genes (*ERG1*, *ERG3*, and *ERG11*), Heat Shock Protein Genes (*HSP12*, *HSP21*, *HSP30*, and *HSP70*), and Agglutinin-like Sequence Families (*ALS2* and *ALS4*)	*C. albicans* (SC5314) ^AR^ *C. albicans* Isolates ^FR^	20 40–80	[75]
*Jeniperus thurifera* L./essencial oil	α-thujene, elemol and muurolol/GC-MS	Unknown	*C. albicans* (ATCC 10231) ^FR^	0.095	[76]
*Sarcococca hookeriana*/extract and fraction	Sarcovagine D/ UPLC-QTOF-MS	Disruption of the Cell Membrane and Biofilms; Interference With the Sphingolipid Pathway.	*C. albicans* (08030401) ^FR^	16	[77]
*Caryocar brasiliense*/extract	Polyphenols/ spectrophotometer	Inhibition of Cell Growth, Damage to the Cell Wall and Plasma Membrane.	*C. albicans* (ATCC 90029) ^5FR^ *C. tropicalis* (ATCC 750) *C. auris* ^FR^ Isolates	32–128	[78]
*Trichilia emetica*/extracts	Gamabufatolin, 9-octadecen-1-ol(Z), octadecanoic acid, rescinnamine, cis-9-tetradecen-1-ol, toluene and 2,4-di-tert-butylphenol/CG-MS	Inhibition of Cell Wall Synthesis and Leakage of Nucleic Acids from the Plasma Membrane	*C. albicans* (NCPF 3255) ^FR^ *C. tropicalis* Isolates	1000 250	[79]

ATCC: American Type Culture Collection; CDC: Centers for Disease Control; CD: Institute’s Adolfo Lutz Culture Collection; NCPF: National Collection of Pathogenic Fungi; National Institute for Quality Control in Health (INCQS); AR: azole-resistant; FR: fluconazole-resistant; RI: intrinsically resistant; ABR: amphotericin B-resistant; CI: clinical isolate; 5FR: 5-flucytosine-resistant; *Candida albicans*: ATCC 10231 (azole-resistant); SC 5314; NCPF 3255; INCQS 40006; 08030401 and ATCC 76645 (fluconazole-resistant); clinical isolate CAAL256 and CA16 (fluconazole-resistant); ATCC 90029 (5-flucytosine-resistant); *Candida krusei*: ATCC 6258 and ATCC 34135 (both intrinsically fluconazole-resistant); INCQS 40095 (fluconazole-resistant); *Candida glabrata*: ATCC 2001 (azole-resistant); *Candida auris*: CDC B11903 and clinical isolate CAAU537 (both resistant to fluconazole and amphotericin B); *Candida tropicalis:* INCQS 40042 (fluconazole-resistant); HR-ESI-MS: high-resolution electrospray ionization mass spectrometry; UPLC-ESI-MS: ultra-performance liquid chromatography–electrospray ionization mass spectrometry; UFLC-QTOF-MS: ultra-fast liquid chromatography–quadrupole-time-of-flight mass spectrometry; GC-MS: gas chromatography–mass spectrometry; UPLC-ESI-QTOF-MS/MS: ultra-performance liquid chromatography coupled with electrospray ionization/quadrupole-time-of-flight-mass spectrometry; HPLC-DAD: high-performance liquid chromatography coupled to diode array detector; NMR: nuclear magnetic resonance; LC-MS QqQ: liquid chromatographic-triple quadrupole tandem mass spectrometry; ESI-MS: electrospray ionization mass spectrometry; LC-ESI-HRMS/MS: liquid chromatography coupled with high-resolution tandem mass spectrometry.
